# A comparative usability assessment of computer input devices for navigating digital whole slide images

**DOI:** 10.1016/j.jpi.2025.100449

**Published:** 2025-05-27

**Authors:** John Rogers, Yuvanesh Vedaraju, Jim Hsu, Jacob Kinskey, S. Wesley Long, Paul Christensen

**Affiliations:** a Houston Methodist Hospital, Department of Pathology and Genomic Medicine, Houston, TX, USA; b Tempus AI Inc, Chicago Illinois, USA

**Keywords:** Whole slide imaging, Digital pathology, Navigation devices, Image management system, Interoperability, Translational research, Clinical informatics

## Abstract

Labs worldwide are increasingly adopting digital pathology due to its ability to facilitate electronic slide distribution and sharing, integration with artificial intelligence tools, and the various workflow improvements enabled by a digital interface. The availability of efficient controls for navigating whole slide images is an important aspect of successful implementation. In this usability study of controller devices, we configured our whole slide image viewer to support navigation using 10 different methodologies, including standard click-and-drag mouse movement, keyboard panning, videogame controllers, and more. Thirty-eight practicing pathologists and trainees volunteered to use these devices and provide feedback. The videogame console gamepad, SpaceMouse Pro, and large trackball emerged as the most preferred devices. After testing each device, 63% of participants indicated that they would need an alternative to standard mouse click-and-drag for effective and efficient case sign-out. These results highlight non-traditional image navigation devices as valuable options in digital pathology implementation and suggest an opportunity for image management systems to differentiate themselves in a competitive marketplace.

## Introduction

Digital pathology is rapidly gaining traction, with the global market growing by 37.3%, 31.4%, and 27.3% over the years 2020–2022, respectively.^1^ The coronavirus disease 2019 (COVID-19) pandemic played a significant role in accelerating the adoption and development of digital pathology due to the increased need for remote work. In parallel, the rising use of artificial intelligence tools has further highlighted the benefits of digital pathology. However, significant barriers to adoption remain, including cost, workflow logistics, and decision paralysis.^2^ Even after an institution commits to implementing digital pathology, a key challenge is the shift in a pathologist's workflow when reviewing slides. Not only are pathologists viewing images on their monitors instead of through microscopes but they must also learn to efficiently navigate both the image management system (IMS) and the digital slide.

Whereas several studies have evaluated whole slide image (WSI) navigation devices,^3^, ^4^, ^5^, ^6^ many were either conducted before the initial adoption of digital pathology for primary diagnosis in the United States or involved a limited number of participants. To better understand WSI navigation devices for primary diagnostic purposes, we piloted 10 devices with 38 practicing pathologists and pathology trainees in the Houston Methodist Department of Pathology and Genomic Medicine. We aimed to determine whether a single preferred device could be identified or if a variety of options would be necessary to meet the preferences of users.

## Materials and methods

This project was approved by the Houston Methodist IRB (PRO00037872) before testing and data collection.

### Configuring RecutClub and the devices tested

**Fig. 1:**
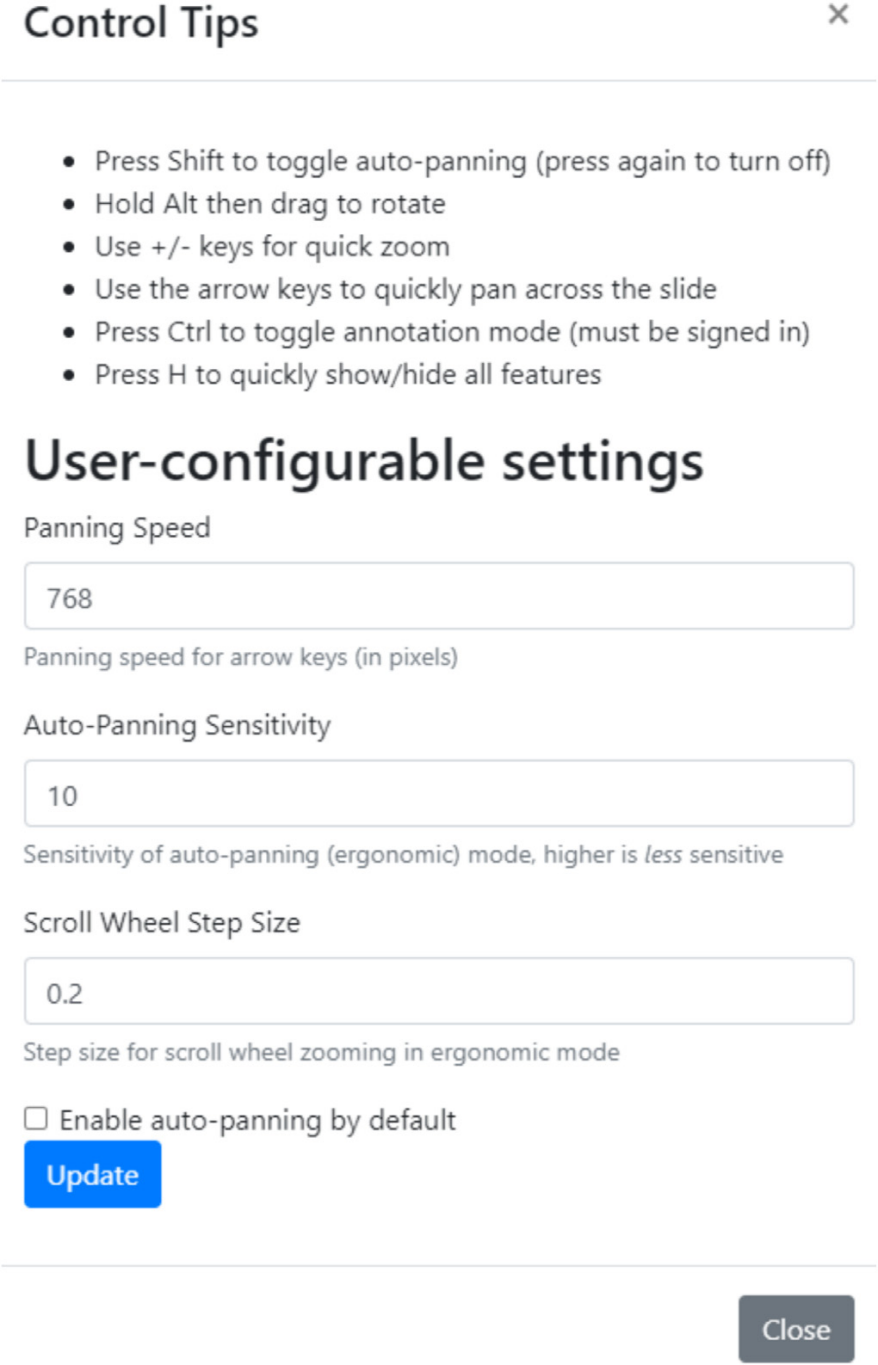
Sensitivity parameters allowing us to customize each device's parameters.

We modified our existing WSI viewer, RecutClub, which is used for resident didactics, to enable navigation of digital slides with new methodologies.^7^ Previously, the RecutClub viewer only supported traditional click-and-drag navigation, along with mouse wheel zooming. For this study, we enhanced the viewer with new navigation tools from the OpenLayers framework (https://openlayers.org/), incorporating keyboard pan, keyboard zoom, and drag rotate. Most importantly, we also implemented a custom auto-panning feature, which allows image panning based on the cursor's distance from the center of the screen. These tools were individually customizable within the viewer (see Fig. 1).

**Fig. 2:**
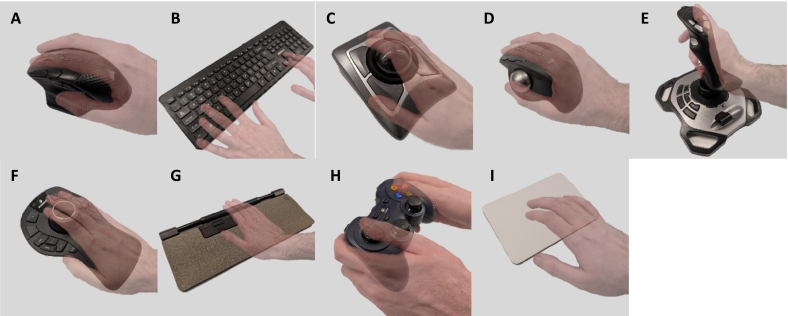
The devices used for navigation testing included a corsair Dark Core RGB Pro Mouse with both click-and-drag and auto-panning (“ergonomic mode”) (A), keyboard (B), Kensington Expert Mouse Trackball (large trackball) (C), Logitech MX ERGO Plus Trackball (thumb trackball) (D), Logitech Extreme 3D Pro Joystick (E), 3DConnexion SpaceMouse Pro (F), Contour Rollermouse Pro (G), Logitech F310 Gamepad (H), and Apple Magic Trackpad (I).

We used these features to test navigation of digital slides with 10 different devices (Fig. 2).

Pan and zoom sensitivities for each device were preset by the research team but could be adjusted by participants during testing.

### Utilizing AutoHotkey scripts to improve auto-panning mode

To facilitate navigation in auto-panning mode, we used AutoHotkey (https://www.autohotkey.com/) scripts to center the cursor after 20 msec of inactivity on the Corsair mouse, large trackball, thumb trackball, SpaceMouse, Rollermouse, and trackpad. Another AutoHotkey script was used to hide the cursor. These scripts were used with a full-screen view of the WSI. When participants pressed the shift key to enable auto-panning, the cursor would disappear, and the image would move based on the hidden cursor. Once movement stopped, the hidden cursor automatically re-centered. These scripts enabled smoother navigation across the WSI for all devices.

### Software and drivers required for other devices

The Logitech joystick and Logitech gamepad were not natively compatible with mouse movements. We used AntiMicroX (https://github.com/AntiMicroX/antimicrox) to map joystick and button inputs to mouse movements and keyboard commands. The sensitivity for slide navigation and zoom was configured in the AntiMicroX profile before testing. The left joystick of the gamepad was mapped to translational movement, whereas the right joystick controlled zoom. The joystick handle was mapped to translational movement, and the hat switch controlled zooming.

The Apple Magic trackpad and 3DConnexion SpaceMouse required additional drivers to enable cursor movement on Windows. We used custom drivers for the Apple Magic Trackpad available on GitHub (https://github.com/imbushuo/mac-precision-touchpad). For the SpaceMouse, we modified the config files to enable “Desktop” mode (https://3dconnexion.com/us/support/faq/can-i-move-the-windows-cursor-with-a-spacemouse/) and allow the device to move the cursor. Zooming was enabled by rotating the SpaceMouse controller clockwise or counterclockwise.

### Method for evaluating devices

After a group orientation where we explained the purpose of the project and obtained informed consent, participants scheduled individual testing sessions lasting 1–2 hr. A member of the research team oversaw each testing session. The testing workstation included a high-resolution ultrawide monitor (LG HDR WQHD monitor with 3440 × 1440 resolution), a wired internet connection with 90 Mbps download speed, an NVIDIA RTX A4000 graphics card, and an Intel i7-12700 processor.

During their assigned sessions, each participant completed a pre-survey to gather pertinent demographic data and assess their assumptions about whether there might be a better alternative to the standard click-and-drag mouse. Participants were provided 20 WSIs to test with each device. The slides covered various subspecialities, including endoscopic biopsies, core needle biopsies, full resections, and cytology specimens. Each participant selected up to two slides to test per device. Participants were encouraged to view these two slides as if they were making a first-time diagnosis. They could either choose to use the same set of two slides across all devices or test different slides for each device. All participants began by testing the standard mouse with click-and-drag functionality. The order of testing for each subsequent device was randomized for each participant. After testing each device, participants completed a survey with feedback specific to that device. Finally, after testing all 10 devices, they filled out a post-testing survey indicating their overall favorite and least favorite devices. Survey design is included in Supplemental Figs. 1–3.

## Results

### Demographic information

Detailed demographic data are shown in Table 1. In total, 38 individuals participated in the study. Most participants were trainees (63%), right-handed (95%), classified themselves as intermediately proficient with computers (66%), and reported using their hand to navigate glass slides (61%). Additionally, before testing, the majority believed that an alternative navigation device would be superior to a click-and-drag interface (92%).

**Table 1:** Demographic data from study participants.

Category	Responses	Number	Percentage
Experience cohort	Trainees	24	63
	Faculty	14	37
Current method of slide navigation	Hand	23	61
	Stage	7	18
	Both	8	21
Dominant hand	Left-handed	2	5
	Right-handed	36	95
Belief in better option	Yes, there is a better option	35	92
	No, click and drag mouse will be the best	3	8
Computer expertise	Novice	8	21
	Intermediate	25	66
	Expert	5	13

### Most preferred devices

Participants rated their overall satisfaction with each device on a scale of 1–5 immediately after testing, as summarized in Table 2.

**Table 2:** Overall satisfaction with each device immediately after testing. Scores reflect user satisfaction on a scale from 1 to 5.

Device	Mean satisfaction score	Median satisfaction score	Standard deviation
Gamepad	4.05	4	1.11
Large trackball	4.03	4	1.10
Thumb trackball	3.61	4	1.41
Joystick	3.58	4	1.13
Click-and-drag mouse	3.58	4	1.20
SpaceMouse Pro	3.53	4	1.22
Rollermouse	3.29	3	1.37
Keyboard	3.03	3	1.37
Trackpad	3.03	3	1.33
Ergonomic mode mouse	2.97	3	1.35

**Fig. 3:**
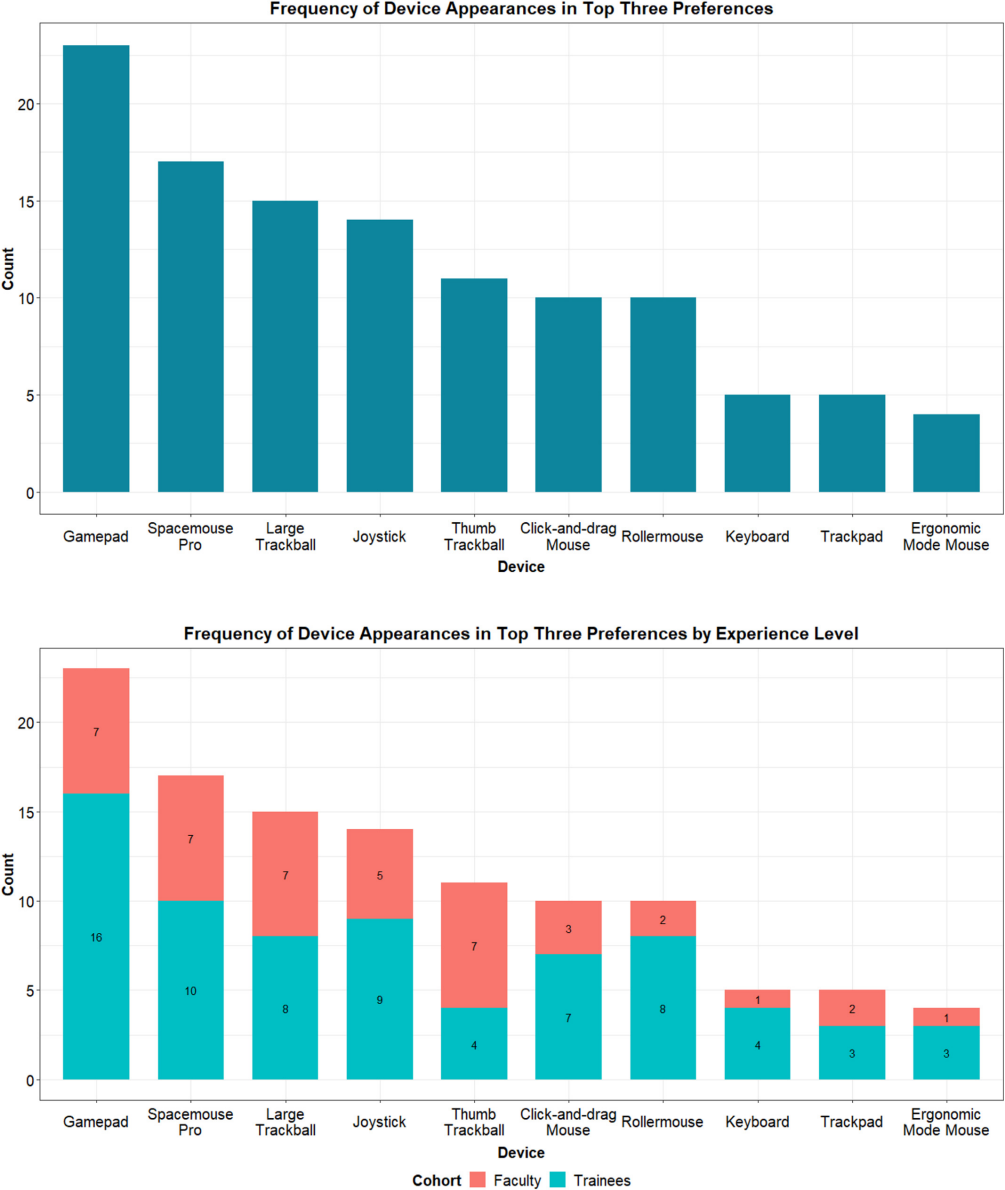

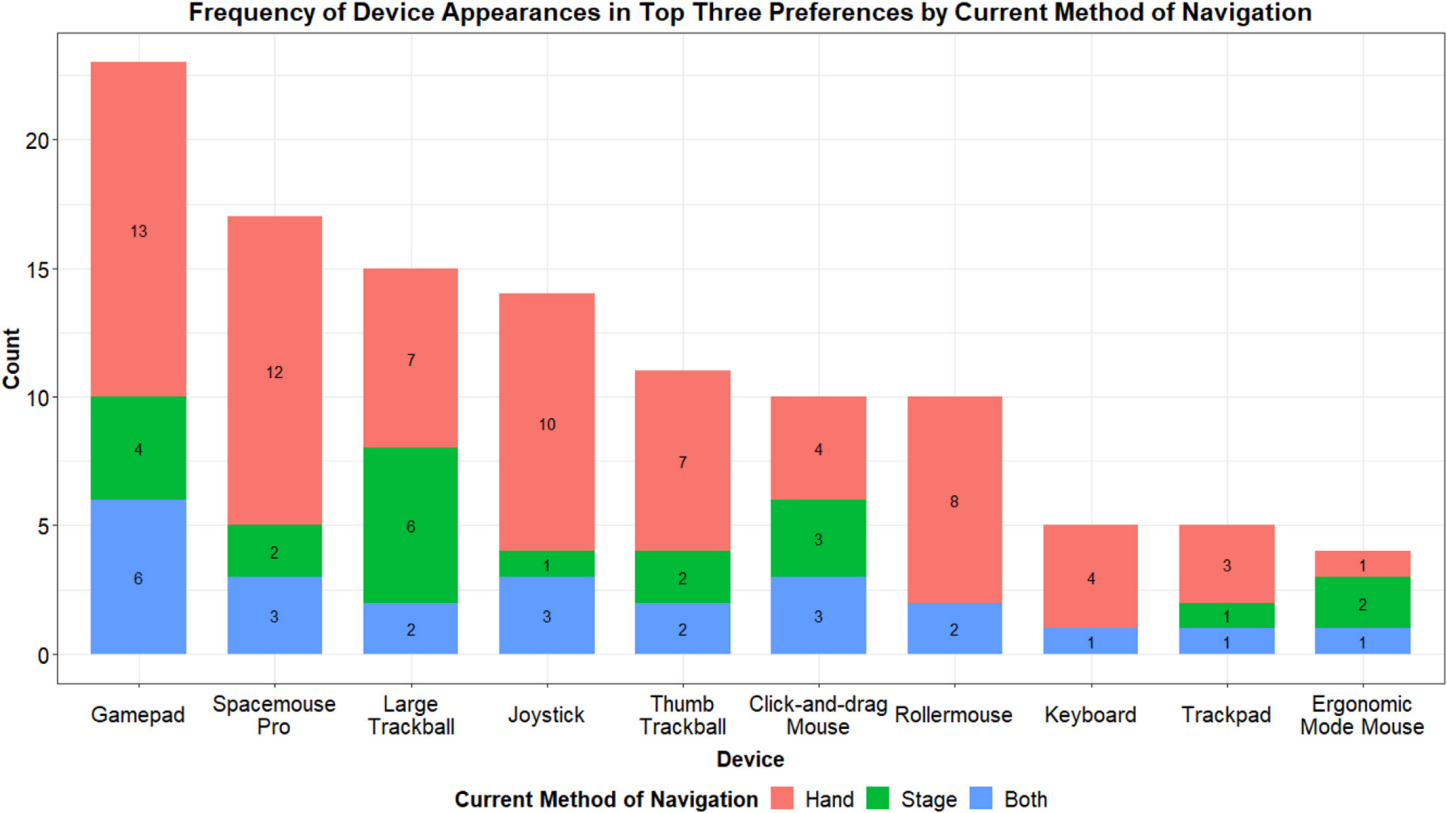
Frequency of device appearances in top three preferences.

At the end of their testing session, participants were asked to list their top three favorite devices, as selecting only one was challenging for participants. Using this method, the gamepad, SpaceMouse Pro, and large trackball emerged as the top three devices preferred by participants. This trend was consistent across different experience levels and methods of glass slide navigation (see Fig. 3).

**Fig. 4:**
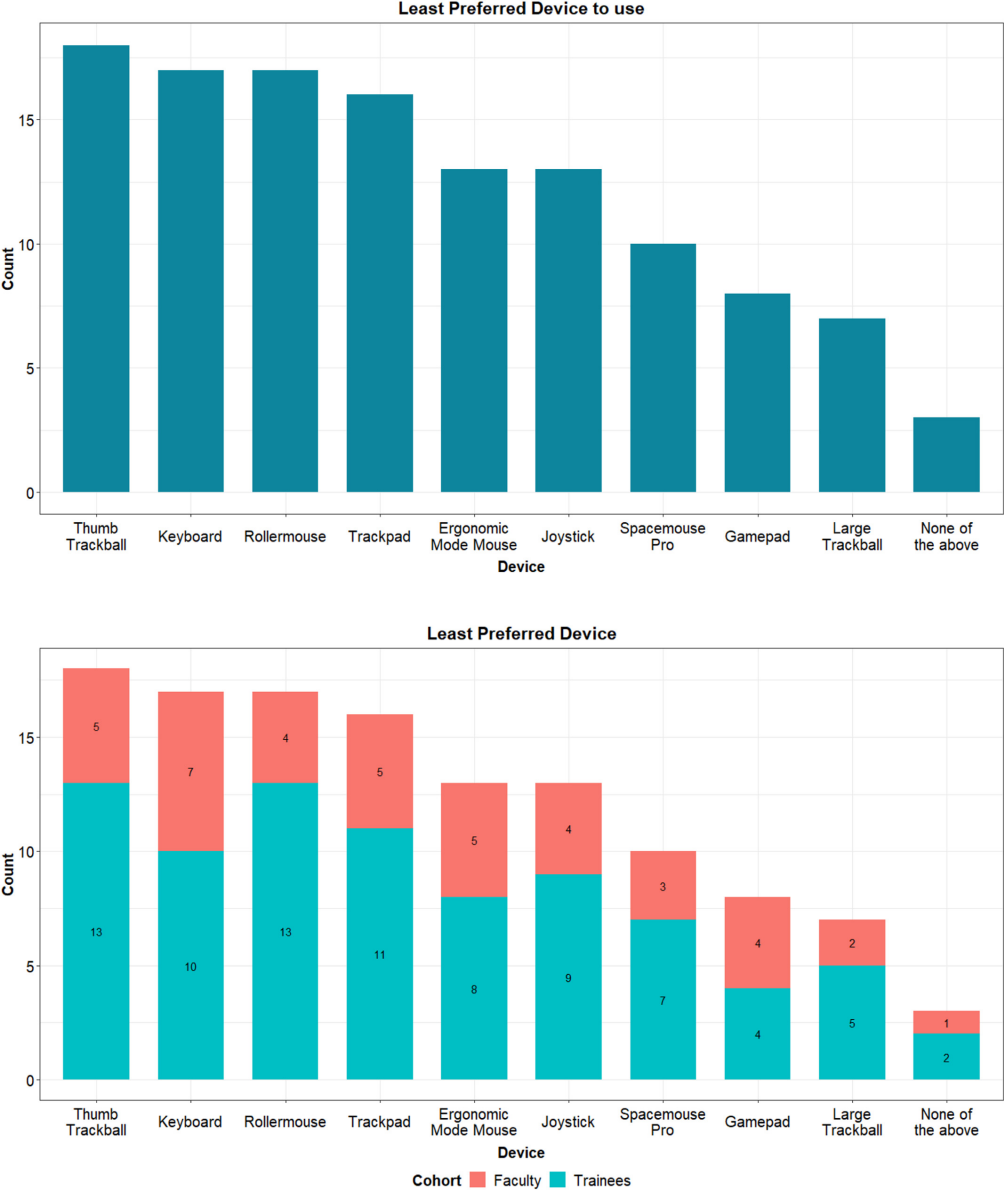

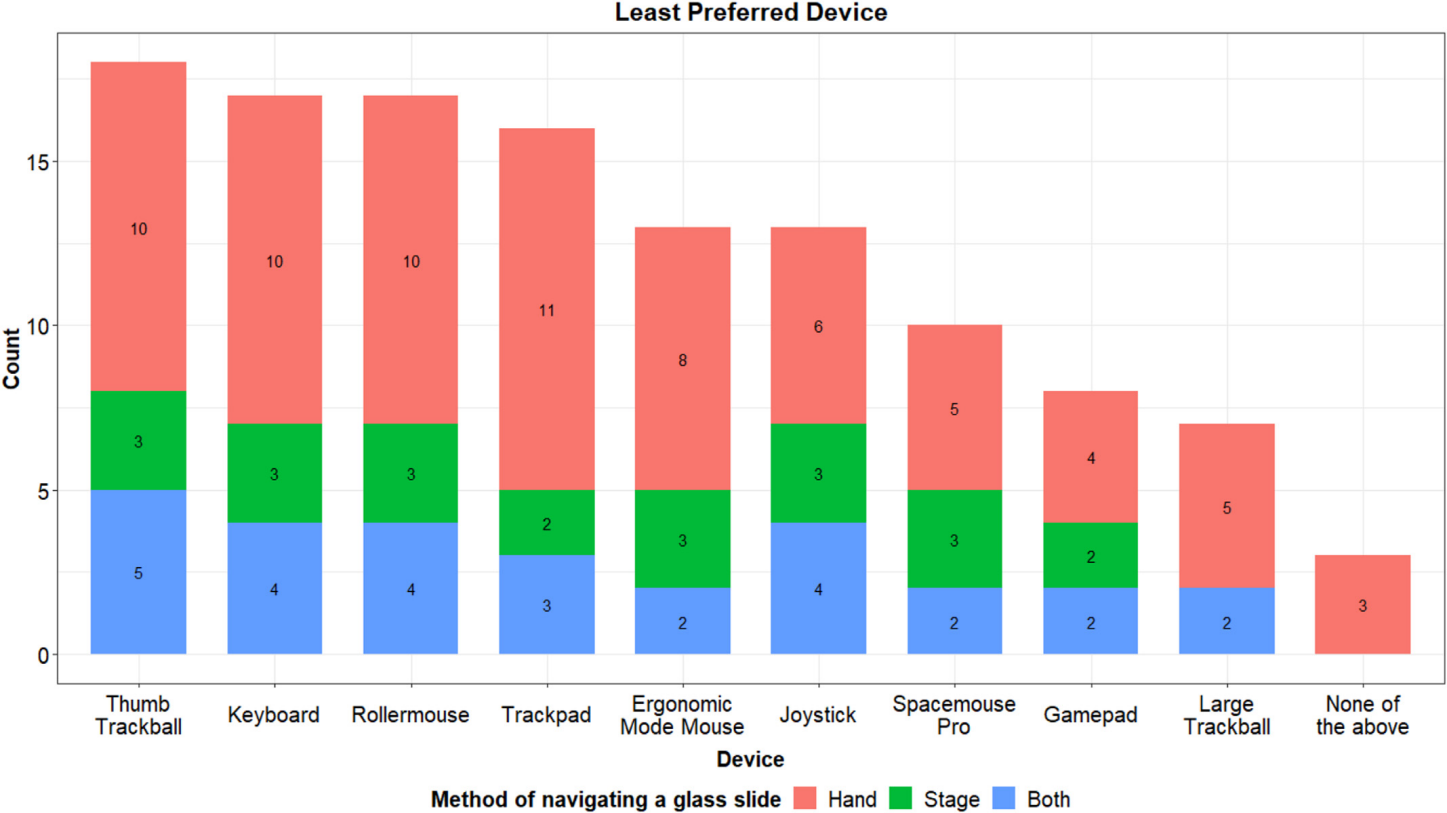
Devices rated as inferior to the click-and-drag interface.

At the end of their testing sessions, participants were asked to list which devices they found inferior to the standard click-and-drag interface. The thumb trackball, keyboard, Rollermouse, and trackpad were the most strongly disliked devices (Fig. 4). This trend was consistent across the training cohort and method of glass slide navigation. Notably, every device was rated at least seven times as being inferior to click-and-drag, with the large trackball being the least disliked.

Finally, 63% of participants indicated they would need an alternate device to maintain their current level of efficiency during case sign-out (see Table 3). Those who felt they could be equally as efficient with a click-and-drag interface cited familiarity, the ability for more accurate and deliberate navigation, and the convenience of not needing another device at their desk. They also felt that other devices did not significantly improve their efficiency. Interestingly, although 37% of participants reported they could sign out efficiently with a click-and-drag mouse, only 26% included it among their top three preferred devices.

**Table 3:** Number of participants reporting they could maintain efficiency using a click-and-drag interface.

Alternate device required to maintain efficiency	Number	Percentage
I need one of the alternate devices to maintain my efficiency	24	63
I could sign out effectively and efficiently with a click-and-drag mouse	14	37

### Regular click-and-drag evaluation

**Fig. 5:**
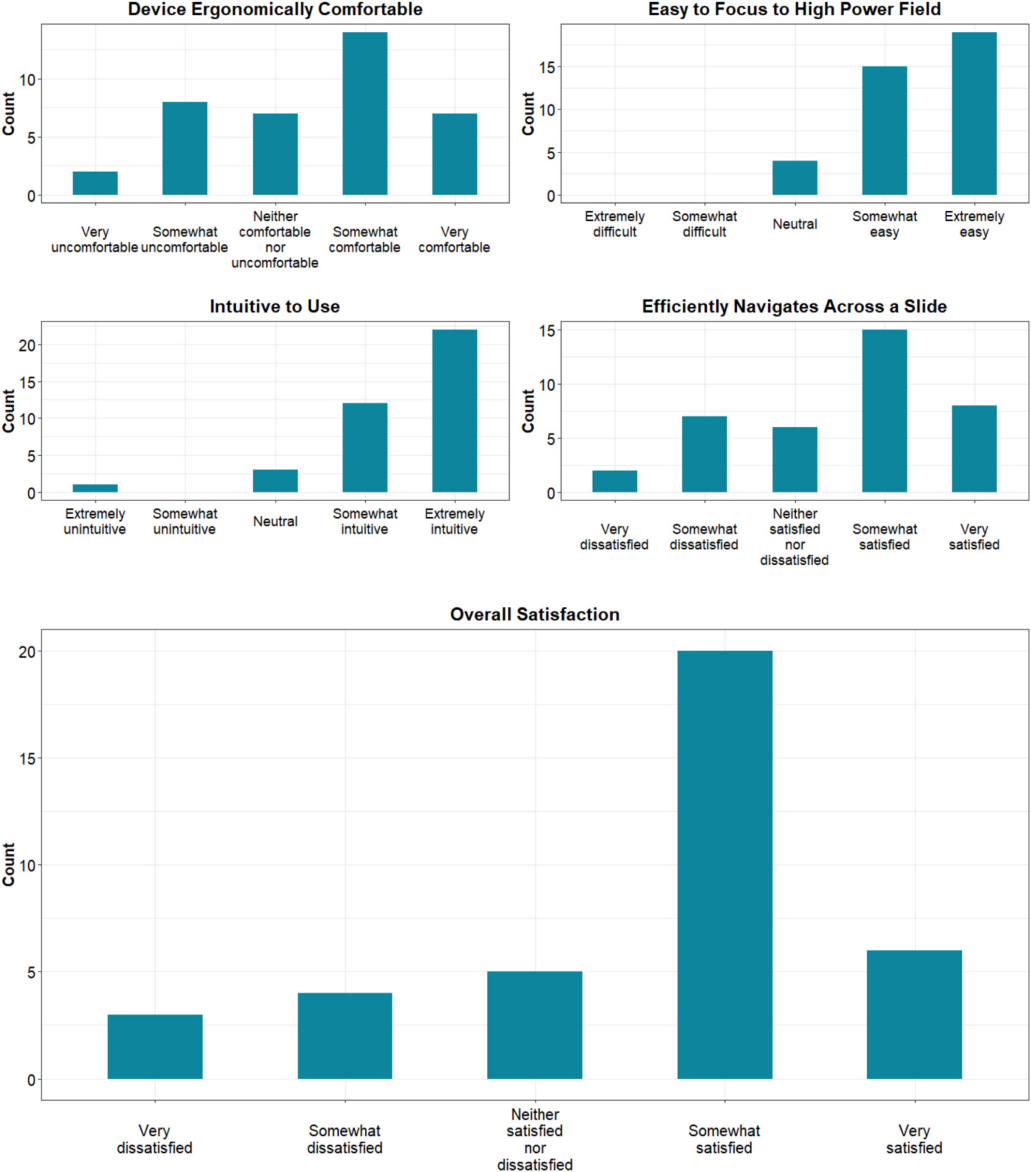
Survey scores for the click-and-drag interface.

The first device tested by each participant was the mouse with a click-and-drag interface, which acted as a “control” device. Trainees and pathologists were accustomed to this method from unknown slide conferences and the Performance Improvement Program by the College of American Pathologists. Overall, participants found the mouse intuitive and easy to focus on areas of interest (Fig. 5). Whereas the ergonomics, efficiency, and overall satisfaction were not rated as highly, the mode for these Likert scale categories remained at 4. Some participants reported that their initial high ratings were influenced by not yet having considered a viable alternative.

### Alternative device evaluations

**Fig. 6:**
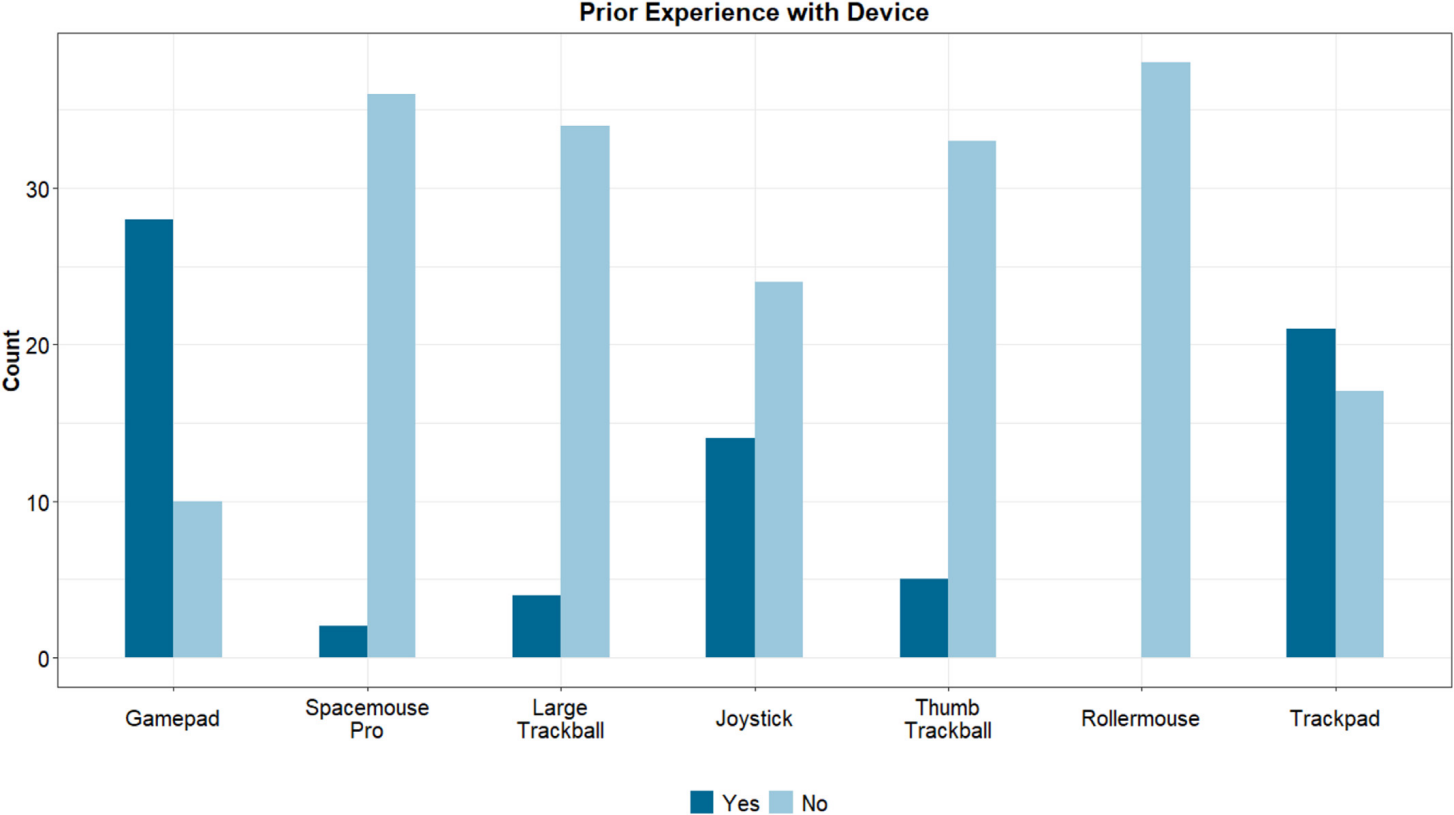
Devices that participants had experience with before the testing session.

**Fig. 7:**
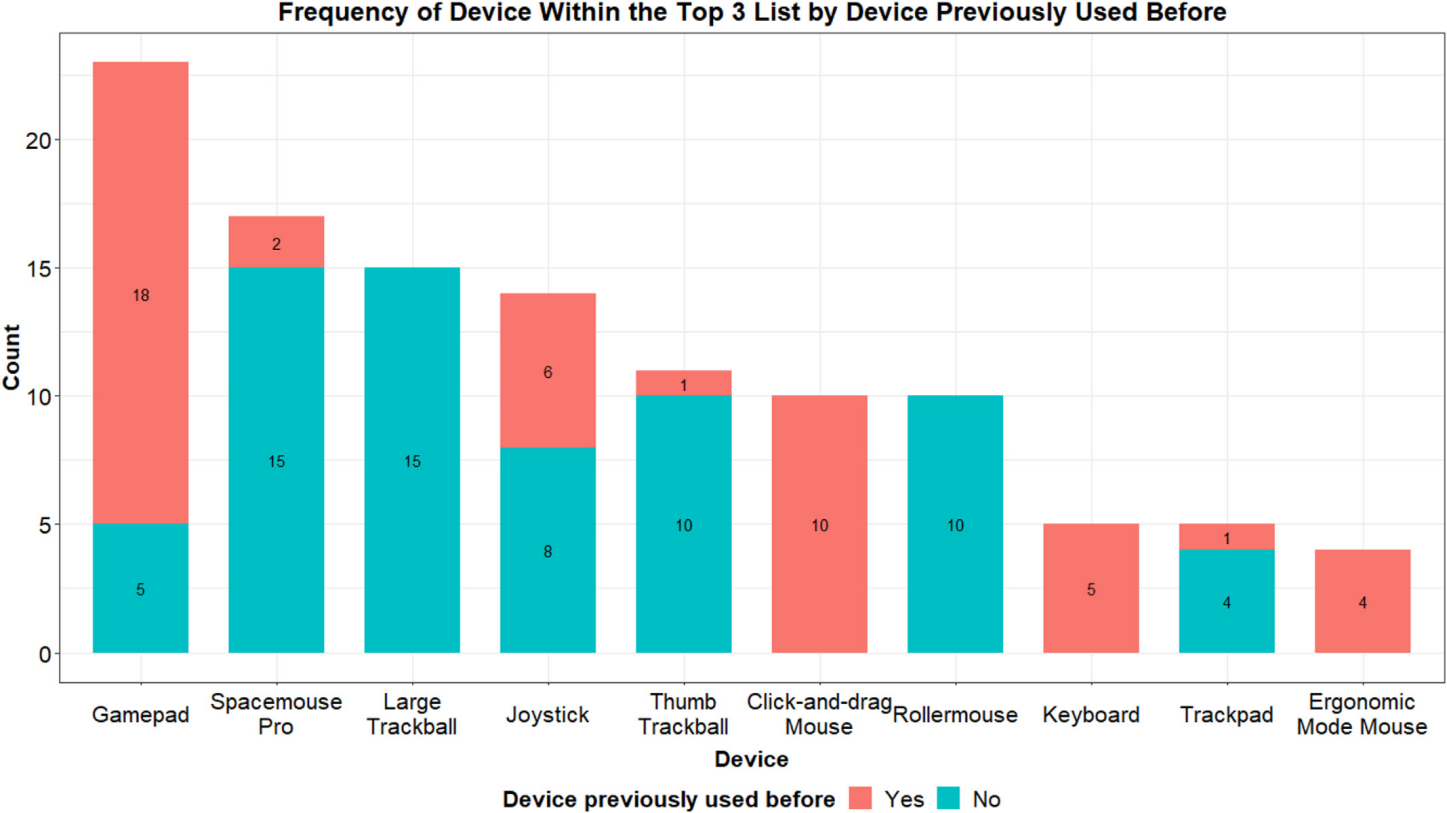
Devices that appeared in the top three list, colored by prior use.

To evaluate whether previous experience with a device influenced participants' preference for certain devices, participants were asked if they had used similar devices before the study (Fig. 6). Except for the regular mouse or keyboard (which all participants had used), the most frequently reported devices were the trackpad, gamepad, and joystick. A small minority had used the SpaceMouse with a previous IMS. A small group of younger participants had prior experience with the thumb trackball and large trackball. No one had previously used the Rollermouse. There was no correlation between previous experience and whether a device ranked in participants' top three list (Fig. 7). Both the SpaceMouse Pro and large trackball, despite being rarely used before, were popular options. The gamepad was the most preferred device and also the most frequently used by participants, possibly due to the larger proportion of younger trainees in the study.

## Discussion

### Most preferred devices

Before testing, the majority believed that an alternative navigation device would be superior to a click-and-drag interface (92%). After testing, the top three devices favored by participants were the gamepad, SpaceMouse Pro, and large trackball. Each device had specific features that made them appealing to participants. The gamepad was familiar to many, allowed for joystick-like navigation without the bulk of a traditional joystick, and allowed users to sit back comfortably in their chairs rather than hunching over a desk. The SpaceMouse was also familiar to some faculty who had used it at other institutions. Like the gamepad, it offered joystick-like functionality. However, we were unable to configure this device to work seamlessly with AntiMicroX and had to use AutoHotkey to continually recenter the cursor. This led to a somewhat jumpy navigation experience, and participants had some difficulty when trying to prevent image movement while zooming by rotating the knob. Despite these challenges, participants appreciated the number of programmable buttons that could be used to disable the rotate-to-zoom feature and streamline their workflow with shortcuts. Finally, the large trackball was surprisingly popular among participants, who reported that it felt the most similar to navigating a glass slide. The main criticism was the ergonomics of the zoom function, which was driven by the ring around the trackball; it was awkward to use and sometimes led to accidental zooming.

### Click-and-drag mouse evaluation

The click-and-drag mouse interface was tested first in every session. Interestingly, despite ranking in the middle of the pack among all devices, participants initially rated it relatively high. The mean satisfaction score was 3.58 on the Likert scale. Despite this, in the exit survey, only 10 participants included it among their top three favorite devices, which placed it sixth overall. Additionally, only 37% of participants believed they could effectively and efficiently navigate with a click-and-drag interface. We believe participants' familiarity with this method of navigating WSIs significantly influenced their evaluations, even if it was not the most efficient option. People tend to prefer familiar workflows, and given the prevalence of the click-and-drag method in online public viewers, it is an initially attractive choice for everyday practice. It remains unclear whether this workflow is ergonomically sustainable over an entire career given the repetitive motions and speed of navigation.

### Reported pros and cons for specific devices

Recurrent comments emerged for each device during testing. Features that made devices appealing included intuitiveness, the ability to move in a grid-like fashion, and the potential to enhance workflow by mapping buttons to shortcuts in the IMS. Criticisms broadly included erratic navigation, lack of ergonomic satisfaction, and excessive demands on fine motor control. For a more detailed explanation of the comments on specific devices, please see Table 4.

**Table 4:** Reported pros and cons for each device.

Device	Pros	Cons
Click-and-drag mouse	1) Intuitive to use	1) Tedious 2) Inefficient for long-term use
Ergonomic mode mouse	1) Intuitive to use	1) Jerky movement 2) Can run out of mousepad space due to inability to reset the mouse
Keyboard	1) Good for grid navigation	1) Too inflexible for diagonal navigation 2) Centering still requires a mouse 3) Jerky movement
Thumb trackball	1) Smooth navigation 2) Zoom is intuitive	1) Causes thumb fatigue 2) Device too large to use effectively
Large trackball	1) Smooth navigation 2) Ambidextrous setup	1) Awkward to use the ring for zoom 2) Trackball too large
Rollermouse	1) Comfortable to use and good for low-power navigation	1) Bar too limiting for navigation 2) Too big to use effectively
Trackpad	1) Smooth zoom	1) Requires repetitive dragging motions 2) Difficult to use 3) Ergonomically uncomfortable
SpaceMouse Pro	1) Intuitive to use 2) Ergonomically comfortable 3) Good workflow potential with many buttons	1) Frequently moves slide while trying to zoom
Joystick	1) Intuitive to use 2) Workflow potential with buttons on the side	1) Stiff to use 2) Ergonomically uncomfortable
Gamepad	1) Intuitive to use 2) Smooth navigation 3) Workflow potential with buttons on the side	1) Requires both hands for navigation

### Anecdotal observations during testing

Several anecdotal behaviors were observed during testing. Some participants found it difficult to replicate their workflow on a WSI like they would on a microscope. They tended to jump to higher power immediately rather than scanning at low power and focusing when needed. This may stem from most participants having limited experience with digital solutions for primary diagnosis and the informal setting of the testing, which occurred outside their normal work environment. Additionally, because most participants had primarily used WSIs for research and taking pictures for publications or tumor boards, they often jumped to high power quickly. Another observation was the wide variation in sensitivity preferences. After 10 participants noted that some default sensitivities were too high, we allowed for individual adjustments, which greatly increased satisfaction with some devices. We invited individuals who tested standard sensitivities to retest, but this did not alter the overall results. Initially, we set higher sensitivity levels assuming that pathologists would prefer to navigate with subtle motions, but a less sensitive approach was preferred by most participants. Finally, the 34-in., 21:9 ultrawide monitor initially felt jarring for some participants. Whereas they appreciated the high-quality resolution of the displayed images, they found the width of the monitor challenging. This ultrawide monitor could be useful for displaying metadata in a viewer, such as gross images, a reporting field, and the section code for the case, but when used solely as a viewer, a 16:9 aspect ratio may be preferred to catch points of interest with peripheral vision.

### Study limitations and future improvements

Our study had three primary limitations. First, our scope was limited to evaluating slide navigation and participants' first impressions of the devices. Second, our participants were predominantly trainees rather than practicing pathologists. Third, there were challenges associated with integrating RecutClub as the image viewer for some of the tested devices.

When designing the study, an early limitation was the lack of an institutionally available IMS to work with. Fortunately, we had a home-grown online viewer, RecutClub, which we used for conferences. However, RecutClub was quite basic and lacked common viewer functionalities such as a thumbnail displaying the current slide position or zoom level. During testing, multiple participants mentioned that having these functionalities would have changed their navigation method. They wanted to use a thumbnail or set hotkeys for specific magnification levels. Whereas RecutClub was sufficient for evaluating the navigation capabilities of each device, its lack of these features led some participants to question whether their dissatisfaction stemmed from the device itself or the limitations of the viewer. Participants also only tested each device for 5–10 min due to time constraints, which likely influenced their initial impressions. Their views on the devices could evolve, an aspect not captured by our study. Another limitation is that when testing 10 different devices, replicating a consistent diagnostic workflow was not possible in the allotted time. This study was designed to gather feedback from many individuals on a broad range of devices. A follow-up study focused on fewer devices and high-volume clinical workflows would be beneficial.

Our participants were recruited internally from the Houston Methodist Department of Pathology and Genomic Medicine through email, and the orientation sessions for the study were held during trainee conference time. As such, most trainees attended one of the two orientation sessions, and a majority elected to participate in the study. Recruiting attending pathologists was more challenging due to the 2-hr time commitment required for testing all the devices and attending the in-person orientation. Clinical volumes also made it difficult for several pathologists, who expressed interest but could not find the time to participate. Trainees, however, had protected conference time each day, which we used for their testing sessions.

Finally, integrating all the devices with RecutClub presented some technical challenges. We realized that navigating the image would require mapping cursor movement relative to the screen center. AntiMicroX provided smooth integration for the gamepad and joystick devices. For other devices, including the SpaceMouse, we required a custom AutoHotKey solution to recenter the cursor after 20 msec of inactivity. This workaround could have contributed to the erratic navigation experienced by some participants. Ideally, a clinical-grade viewer from a dedicated WSI vendor would have optimized solutions for integrating these devices.

### Future testing of natural user interfaces

Going beyond standard input devices, natural user interfaces (NUIs) have been tested in both digital pathology and other clinical areas.^8^^,^^9^ In these studies, there have been perceived issues in adoption including software limitations, fatigue, and frustration due to the devices being unintuitive. In the study performed by Alcarez-Mateos et al., they found that using these devices did not significantly impact time to report cases compared with averages, though there were frustrations with adapting to the technology. In Cronin and Doherty's review, they point out the necessity for ample space when using gesture controls and voice controls being difficult due to accents and ambient noise. We believe that NUIs show practical benefit in reducing physical fatigue, but the adoption of these devices may be slow because of the dramatic paradigm shift in signing out with digital pathology. They may be seen as one additional thing a pathologist has to learn and overwhelm them. Incorporating these devices after digital pathology is fully into the workflow may be a more realistic option.

## Conclusion

### Local implications

The results of this study will inform our strategy for implementing digital pathology at our institution. Before participating in the study, pathologists reported that navigating digital slides was an aspect of digital pathology they had not considered in detail. Most assumed that a click-and-drag interface would be the default, without alternate options. Whereas some devices stood out as favorites for individuals, there was a range of preferences across the spectrum of devices. During testing, pathologists also raised concerns about the potential inconvenience of needing an additional device on their desk while still frequently using the keyboard and regular mouse. An IMS with integrated navigational shortcuts compatible with multiple devices may therefore be preferred.

### Importance of testing before adopting digital pathology

Transitioning to a digital workflow is a monumental process with many moving parts. One often overlooked component is re-learning how to navigate a digital slide. A digital interface offers many advantages, such as overlaying slides for immunohistochemistry comparisons, incorporating machine learning models into routine workflows, integrating metadata, and much more. However, at the most basic level, a pathologist still needs to navigate the slide efficiently and effectively. Before this study, our research team strongly disliked navigating with the large trackball; however, during testing, many participants favored it. This further emphasizes the importance of involving local pathologists early in the adoption process and addressing basic questions. Engaging pathologists makes the adoption process collaborative, familiarizes them with the IMS, and should lead to greater satisfaction with the digital pathology workflow.

## Declaration of competing interest

The authors declare that they have no known competing financial interests or personal relationships that could have appeared to influence the work reported in this article.

## Appendix A Supplementary data

Supplementary material Detailed Survey Questionnaire Supplementary material
