# Supplementary material for: A comparative usability assessment of computer input devices for navigating digital whole slide images
Source: J Pathol Inform. 2025 May 27;18:100449. doi: 10.1016/j.jpi.2025.100449 (PMC12221461; doi:10.1016/j.jpi.2025.100449)
Supplement: Supplementary material — Detailed Survey Questionnaire [file mmc1.docx]

# **Supplemental Figures**

## *Supplemental Figure 1: Demographic survey*

1. What is your level of training or how many years have you been practicing as an attending?
   1. PGY1
   2. PGY2
   3. PGY3
   4. PGY4
   5. PGY5
   6. PGY6
   7. PGY7
   8. PGY8
   9. 0-5 years of practice as an attending
   10. 6-10 years of practice as an attending
   11. >10 years of practice as an attending
2. Do you primarily drive with a stage, your hand, or use both interchangeably?
   1. Stage
   2. Hand
   3. Both
3. Are you primarily right or left-handed?
   1. Right
   2. Left
4. Would you classify yourself as a novice, intermediate, or expert user when it comes to computers?
   1. Novice
   2. Intermediate
   3. Expert
5. Do you believe there is a better option for navigating a whole slide image other than standard mouse click and drag?
   1. Yes, there is a better option
   2. No, click and drag will be the best

## *Supplemental Figure 2: Device survey*

1. What device did you evaluate?
   1. Regular mouse without ergonomic mode
   2. Regular mouse with ergonomic mode
   3. Keyboard
   4. Trackpad
   5. Thumb trackball
   6. Large trackball
   7. Rollermouse
   8. SpaceMouse Pro
   9. Joystick
   10. Gamepad
2. Have you ever used this device or something similar routinely before?
   1. Yes
   2. No
3. What slides did you evaluate the devices with?
   1. Slide 1
   2. Slide 2
   3. Slide 3
   4. Slide 4
   5. Slide 5
   6. Slide 6
   7. Slide 7
   8. Slide 8
   9. Slide 9
   10. Slide 10
   11. Slide 11
   12. Slide 12
   13. Slide 13
   14. Slide 14
   15. Slide 15
   16. Slide 16
   17. Slide 17
   18. Slide 18
   19. Slide 19
   20. Slide 20
4. How ergonomically comfortable is this device to use?
   1. 5- Very comfortable
   2. 4 - Somewhat comfortable
   3. 3- Neither comfortable nor uncomfortable
   4. 2 - Somewhat uncomfortable
   5. 1 - Very uncomfortable
5. How easy was it to focus to high power in an area of interest?
   1. 5 - Extremely easy
   2. 4 - Somewhat easy
   3. 3 – Neutral
   4. 2 - Somewhat difficult
   5. 1 - Extremely difficult
6. Overall, how intuitive was the device to use?
   1. 5 - Extremely intuitive
   2. 4 - Somewhat intuitive
   3. 3 – Neutral
   4. 2 - Somewhat unintuitive
   5. 1 - Extremely unintuitive
7. How satisfied were you with the overall speed to navigate across the whole slide image?
   1. 5 - Very satisfied
   2. 4 - Somewhat satisfied
   3. 3 - Neither satisfied nor dissatisfied
   4. 2 - Somewhat dissatisfied
   5. 1 - Very dissatisfied
8. What is your overall satisfaction with the device for navigating a whole slide image?
   1. 5 - Very satisfied
   2. 4 - Somewhat satisfied
   3. 3 - Neither satisfied nor dissatisfied
   4. 2 - Somewhat dissatisfied
   5. 1 - Very dissatisfied
9. Do you have any positive feedback for the device?
   1. Free text
10. Do you have any additional criticisms for the device?
    1. Free text

## *Supplemental Figure 3: Post-testing survey*

1. Did you prefer click-and-drag mouse movement to any of the devices?
   1. Regular Mouse with Ergonomic Mode
   2. Keyboard
   3. Trackpad
   4. Thumb trackball
   5. Large trackball
   6. Rollermouse
   7. SpaceMouse Pro
   8. Joystick
   9. Gamepad
2. What were your top three favorite devices?
   1. Regular Mouse without Ergonomic Mode
   2. Regular Mouse with Ergonomic Mode
   3. Keyboard
   4. Trackpad
   5. Thumb trackball
   6. Large trackball
   7. Rollermouse
   8. SpaceMouse Pro
   9. Joystick
   10. Gamepad
3. Of these three devices, which was your favorite to use?
   1. Regular Mouse without Ergonomic Mode
   2. Regular Mouse with Ergonomic Mode
   3. Keyboard
   4. Trackpad
   5. Thumb trackball
   6. Large trackball
   7. Rollermouse
   8. SpaceMouse Pro
   9. Joystick
   10. Gamepad
4. Why was this device your favorite to use?
   1. Free text
5. Would you be able to effectively and efficiently sign out cases with a standard click and drag mouse or would you require one of the alternate devices you tested today?
   1. Yes, I could sign out effectively and efficiently with a click and drag mouse
   2. No, I need one of the alternate devices to maintain my efficiency
